# External Workload Can Be Anticipated During 5 vs. 5 Games-Based Drills in Basketball Players: An Exploratory Study

**DOI:** 10.3390/ijerph17062103

**Published:** 2020-03-22

**Authors:** Cody J. O’Grady, Vincent J. Dalbo, Masaru Teramoto, Jordan L. Fox, Aaron T. Scanlan

**Affiliations:** 1 School of Health, Medical and Applied Sciences, Central Queensland University, Rockhampton, QLD 4702, Australia; 2 Human Exercise and Training Laboratory, Central Queensland University, Rockhampton, QLD 4702, Australia; 3 Division of Physical Medicine and Rehabilitation, University of Utah, Salt Lake City, UT 84108, USA

**Keywords:** microsensor, accelerometer, PlayerLoad, team sport, small-sided games

## Abstract

This study determined whether external workload could be anticipated during 5 vs. 5 games-based drills in basketball. Thirteen semi-professional, male basketball players were monitored during 5 vs. 5 training drills across the season. External workload was determined using PlayerLoad™ (AU∙min^−1^). The reference workload for each drill was calculated across all sessions, using bootstrapping. The bootstrap mean workload and 95% confidence intervals (CI) were then calculated for session 1, sessions 1–2, and continued for remaining sessions (1–3, 1–4, etc.), and were compared with those of the reference workload. The minimum sessions to anticipate workload for each drill was identified when the first normative value fell within ±5% or ±10% of the reference workload 95% CI. The minimum sessions were then tested to determine the accuracy to which workload could be anticipated. Three to four sessions were needed to anticipate workload within ±5%, while 2–3 sessions were needed to anticipate workload within ±10%. External workload was anticipated in 0–55% of future sessions using an error range of ±5%, and in 58–89% of sessions using an error range of ±10%. External workload during 5 vs. 5 games-based drills can be anticipated in most sessions using normative values established during a short-term monitoring period with an error range of ±10%.

## 1. Introduction

Basketball is a highly intermittent team sport, with players performing repeated bouts of high-intensity activity interspersed with periods of low- to moderate-intensity activity [1,2]. Although the activities undertaken during these periods vary in terms of movement pattern, intensity, and duration, basketball players are required to perform extensive sprinting and high-intensity shuffling activity highlighting the need to perform repeated maximum efforts during game-play [1,2] Quantifying and managing player workloads is therefore essential and enables basketball coaches to prescribe training and recovery plans that best promote favorable performance while reducing the likelihood of maladaptive responses such as illness, injury, and overtraining [3]. Player workloads can be categorized as external, representing the physical stimuli imposed, or internal, representing the psycho-physiological response of players to the external stimuli [4]. Although it is beneficial to quantify external and internal workloads in combination, the external workload is manipulated by basketball coaches to elicit the desired responses in players [4]. Consequently, it is important for external workloads to be accurately prescribed and quantified as players may be susceptible to negative consequences if insufficient or excessive demands are experienced.

Monitoring players during both training and games is preferred to understand the complete external workloads encountered. However, monitoring external workload is more common in training than games possibly due to player preferences and restrictions in some leagues prohibiting use of wearable technologies during games [5]. It is also difficult to attain normative external workload values for games given many contextual factors promote variability in the game-to-game demands encountered by players including game location (home or away) [6], game outcome (win or loss) [6], score-line margin (balanced or unbalanced) [6], and game schedule [7]. Due to the difficulties in obtaining and interpreting external workload consistently during games, it is particularly important for basketball coaching staff to monitor players during training and consider these data when prescribing or manipulating the workloads administered across the season.

Games-based drills are an effective training strategy frequently used by basketball coaches [8,9]. In fact, games-based drills represent a major component of the training process in basketball teams as a means to deliver sport-specific physical, physiological, and tactical stimuli in the training environment [8,9]. In this regard, 5 vs. 5 games-based drills are of particular interest to coaches given they involve team sizes used during competitive game-play [10]. As a result, 5 vs. 5 games-based drills provide stimuli that closely mimic game-specific movement patterns, while developing decision-making and problem-solving skills indicative of competitive settings [9]. Given the benefits of 5 vs. 5 games-based drills in replicating game scenarios, it is important for coaches to understand the external workloads elicited in different 5 vs. 5 formats so appropriate stimuli are delivered to players.

Microsensor monitoring systems encompassing tri-axial accelerometers, gyroscopes, magnetometers, or a combination of these instruments [11], are commonly used to objectively measure external workload in basketball [12,13,14]. Microsensors are appealing in basketball given the associated use of proprietary software for efficient processing and analysis of external workload data [15], as well as reduced potential for human error and ambiguity around interpretation of activity that exists with other approaches such as video-based time-motion analysis [16]. However, few studies [10,17,18,19,20] have quantified the external workloads experienced during basketball games-based drills using microsensors with even less work examining 5 vs. 5 game formats [10,17,18,19]. The limited number of studies reporting external workload during games-based drills using microsensors is likely a function of the long-term costs associated with use of microsensor hardware and software. Consequently, these limitations may be overcome if basketball coaches could anticipate the external workload experienced by players following a short monitoring period to establish normal values, potentially increasing the feasibility of microsensor monitoring systems for teams. Moreover, the ability to anticipate the external workloads encountered by players may allow basketball coaches to be proactive in prescribing games-based drills during training rather than retrospectively reviewing player workload data following sessions to confirm the demands encountered. Consequently, anticipation of normal external workload values for different games-based drills would likely enable basketball coaches to prescribe and periodize workloads across the season with optimal precision. Therefore, the aims of this study are to: (1) Quantify the external workload experienced by players during 5 vs. 5 games-based drills and (2) determine if the external workload encountered during 5 vs. 5 games-based drills can be anticipated across the season.

## 2. Materials and Methods

### 2.1. Subjects

Thirteen players (age: 21.4 ± 4.2 years, stature: 189.5 ± 7.4 cm, body mass: 85.2 ± 19.3 kg) from the same semi-professional, male basketball team volunteered to participate in this study. The team was registered in the Queensland Basketball League, which is a second-tier, Australian basketball competition. Prior to study commencement, all participants were screened for injuries or health conditions that prevented safe participation in the study. The purpose of the study as well as associated risks and benefits of participation were explained to participants before providing personal and guardian written informed consent (if under 18 years). All procedures were approved by the Central Queensland University Human Research Ethics Committee (reference number: 2019-001).

### 2.2. Design

An observational study design was utilized whereby players were monitored during all training sessions across the 16-week, in-season phase (May–August) of the 2019 competitive season. During the season, up to 3 training sessions (2 ± 1 training sessions) were held per week (Monday–Sunday) with games played between Friday–Sunday. A total of 26 on-court, team training sessions were held across the season.

### 2.3. Procedures

Prior to commencement of the first training session, anthropometric data were collected on each player including stature using a portable stadiometer (Seca 213, Seca GMBH, Hamburg, Germany) and body mass using electronic scales (BWB-600, Tanita Corporation, Tokyo, Japan). For all training sessions, players were fitted with microsensors (OptimEye s5, Catapult Innovation, Melbourne, Australia) held on the posterior, upper surface of the torso in specially-designed neoprene shirts. All players were familiar with and accustomed to the equipment used in the present study.

During each training session and following a standardized warm-up, games-based drills were conducted as part of the normal training regime determined by the coaching staff. Three distinct 5 vs. 5 drills were frequently performed by the team and thus were selected for investigation, including: (1) 5 vs. 5 played on a half-court, (2) 5 vs. 5 played on a full-court, and (3) 5 vs. 5 vs. 5 played on a full-court. Each of these drills were performed toward the end of each training session at similar time-points (5 vs. 5 half-court: 49.6 ± 14.6 min into the training session; 5 vs. 5 full-court: 55.9 ± 18.8 min into the training session; 5 vs. 5 vs. 5; 40.3 ± 10.6 min into the training session) following standardized shooting drills and initial games-based drills with less players (e.g., 3 vs. 3). The games-based drills investigated along with associated rules and conditions are presented in Table 1.

Following each training session, microsensor data were downloaded to a personal computer for further analysis using proprietary software (OpenField version 8, Catapult Innovations, Melbourne, Victoria, Australia). External workload was determined via PlayerLoad™ (PL) (Catapult Innovations, Melbourne, Victoria, Australia), which is a proprietary measure derived from the tri-axial accelerometer sampling at 100 Hz. PL represents the accumulated load calculated as the square root of the sum of the squared rate of change in acceleration across the transverse (*x*), coronal (*y*), and sagittal (*z*) planes multiplied by a scaling factor of 0.01 [17]. All activity from the start to finish of each drill was included for analysis. PL was reported in arbitrary units relative to drill duration (AU∙min^−1^) to account for the varied durations of each drill across the season (Table 1). Where a drill was performed more than once within a given session, only the first occurrence of the drill was utilized in the analyses to control for the confounding effects of fatigue on the workloads experienced by players. In this regard, 5 vs. 5 half- and full-court drills were performed more than once in 21% and 47% of training sessions, respectively, while 5 vs. 5 vs. 5 drills were performed more than once in 67% of training sessions. The reliability of PL has been previously supported in court-based, team sports [21].

### 2.4. Statistical Analyses

A two-step approach was adopted to determine if external workload for 5 vs. 5 games-based drills could be accurately anticipated from completed 5 vs. 5 games-based drills. The first step involved identifying the minimum number for sessions required to establish a reference (normal) external workload for each drill. Participation in drills were not consistent for each player across all training sessions. Further, the sample size of the study was not large. Hence we performed nonparametric bootstrapping (1000 replications) to better estimate mean workload values and a bias-corrected 95% confidence interval (CI) for each mean workload value [22,23]. A bootstrapping approach was used to better estimate the variability and 95% confidence intervals for the outcome variables, since the sample size in this study was not large. Further, bootstrapping allowed us to use the empirical distributions of the outcome variables instead of relying on the theoretical distributions [24]. First, to establish the reference workload, the bootstrap mean with bias-corrected 95% CI of the average workload across all training sessions was calculated. Then, the bootstrap workload mean with 95% CI in session 1 was calculated. Following this, the bootstrap workload mean with 95% CI for the average workload for sessions 1 and 2 combined (sessions 1–2) was calculated. This approach was continued until the bootstrap workload mean with 95% CI across all completed sessions were calculated (i.e., sessions 1–3, sessions 1–4). We then compared the 95% CI for each workload mean (i.e., session 1, sessions 1–2, sessions 1–3, sessions 1–4) with the 95% CI of the reference workload (calculated across all sessions). Given the exploratory and applied nature of this study, we set two separate a priori acceptable error ranges, 5% and 10%, to inform end-users on the ability to anticipate external workload depending on their pre-determined error limits. The minimum number of sessions required to anticipate workload for each drill was identified as the first instance where the 95% CI of the mean workload was within ±5% or ±10% of the 95% CI of the reference workload.

The second step involved testing the accuracy of the anticipated external workload in each drill across subsequent training sessions. We compared the 95% CI of each mean workload identified in the first step of our analyses for each drill to the 95% CI of the bootstrap mean workload experienced in each subsequent individual session. In this regard, if the 95% CI for workload in each drill in a specific session was within the acceptable error range (±5% or ±10% analyzed separately) of the 95% CI of the workload corresponding to the minimum number of sessions identified, workload for that session was adjudged to be accurately anticipated. All statistical analyses were conducted using Stata/MP 16.0 for Windows (StataCorp LLC, College Station, TX, USA).

## 3. Results

**Figure 1:**
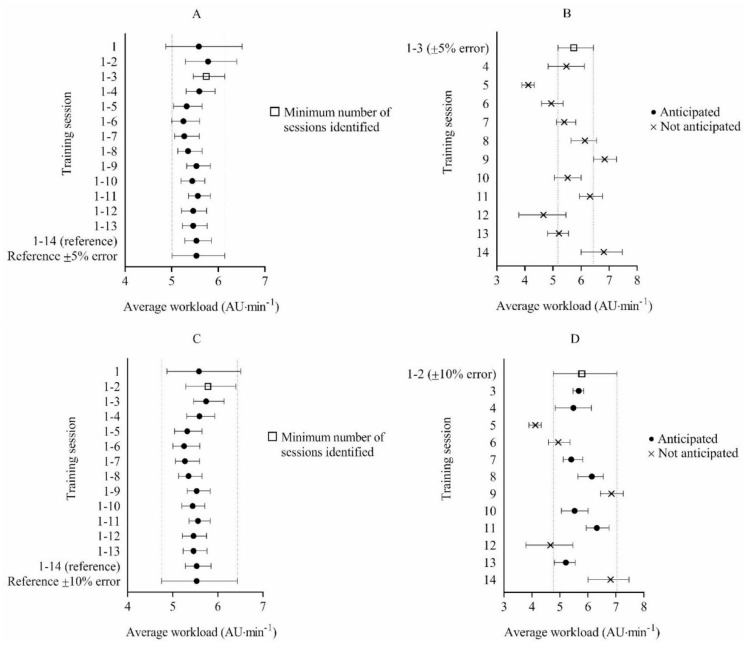
Bootstrap mean workload and bias-corrected 95% confidence intervals (CI) for determining the minimum number of sessions required to anticipate external workload with an acceptable error range of ±5% (**A**) and ±10% (**C**) during 5 vs. 5 half-court games-based drills. Bootstrap mean workload and bias-corrected 95% CI for each future session and the accuracy of anticipating each session using the mean workload from sessions 1–3 with a ±5% acceptable error range (**B**) and sessions 1–2 with a ±10% acceptable error range (**D**) during 5 vs. 5 half-court games-based drills.

Regarding 5 vs. 5 half-court games-based drills, 14 training sessions were included for analysis and used to establish the reference workload. The reference workload was 5.53 AU·min^−1^, 95% CI = 5.28, 5.85. The minimum number of sessions needed to anticipate the workload per session within ±5% for 5 vs. 5 half-court games-based drills was 3 (5.74 AU·min^−1^, 95% CI = 5.46, 6.14), while the minimum number of sessions needed to anticipate workload within ±10% was 2 (5.78 AU∙min^−1^, 95% CI = 5.29, 6.40). Using the acceptable error range of ±5%, mean workload from sessions 1–3 did not accurately anticipate the experienced workload in any (0%) remaining sessions. Using the acceptable error range of ±10%, mean workload from sessions 1–2 accurately anticipated the experienced workload in 58% of remaining sessions (Figure 1).

**Figure 2:**
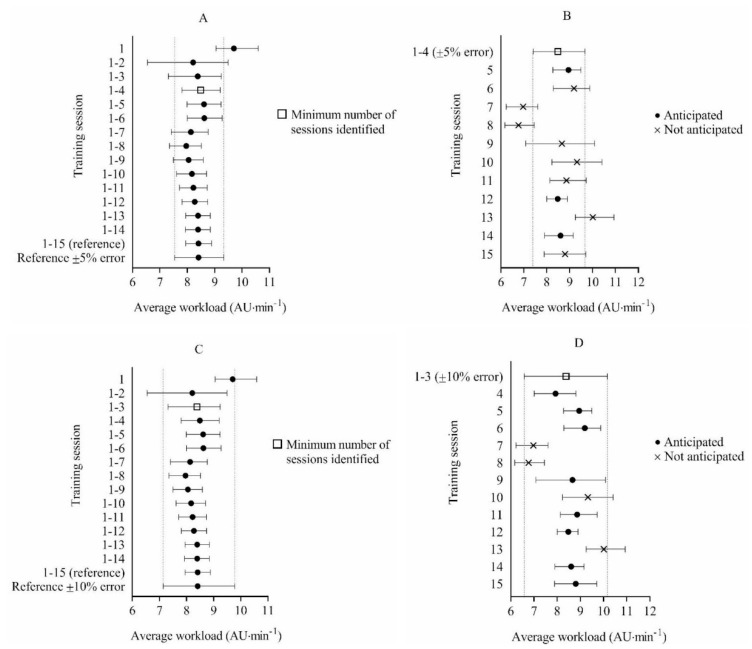
Bootstrap mean workload and bias-corrected 95% confidence intervals (CI) for determining the minimum number of sessions required to anticipate external workload with an acceptable error range of ±5% (**A**) and ±10% (**C**) during 5 vs. 5 full-court games-based drills. Bootstrap mean workload and bias-corrected 95% CI for each future session and the accuracy of anticipating each session using the mean workload from sessions 1–4 with a ±5% acceptable error range (**B**) and sessions 1–3 with a ±10% acceptable error range (**D**) during 5 vs. 5 full-court games-based drills.

For 5 vs. 5 full-court games-based drills, 15 training sessions were included for analysis and used to establish the reference workload. The reference workload was 8.41 AU·min^−1^, 95% CI = 7.94, 8.88. The minimum number of sessions needed to anticipate the workload per session within ±5% for 5 vs. 5 full-court games-based drills was 4 (8.49 AU·min^−1^, 95% CI = 7.80, 9.20), while the minimum number of sessions needed to anticipate workload within ±10% was 3 (8.38 AU∙min^−1^, 95% CI = 7.31, 9.24). Using the acceptable error range of ±5%, mean workload from sessions 1–4 accurately anticipated the experienced workload in 27% of remaining sessions. Using the acceptable error range of ±10%, mean workload from sessions 1–3 accurately anticipated the experienced workload in 67% of remaining sessions (Figure 2).

**Figure 3:**
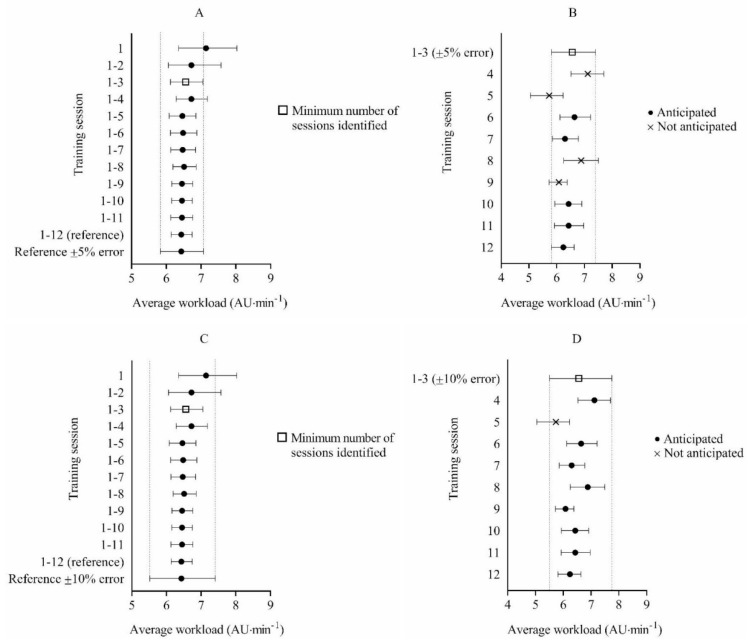
Bootstrap mean workload and bias-corrected 95% confidence intervals (CI) for determining the minimum number of sessions required to anticipate external workload with an acceptable error range of ±5% (**A**) and ±10% (**C**) during 5 vs. 5 vs. 5 games-based drills. Bootstrap mean workload and bias-corrected 95% CI for each future session and the accuracy of anticipating each session using the mean workload from sessions 1–3 with a ±5% acceptable error range (**B**) and sessions 1–3 with a ±10% acceptable error range (**D**) during 5 vs. 5 vs. 5 games-based drills.

Regarding 5 vs. 5 vs. 5 games-based drills, 12 training sessions were included for analysis and used to establish the reference workload. The reference workload was 6.43 AU·min^−1^, 95% CI = 6.14, 6.74. The minimum number of sessions needed to anticipate the workload per session within ±5% and ±10% for 5 vs. 5 vs. 5 games-based drills was 3 (6.56 AU·min^−1^, 95% CI = 6.12, 7.05). Using the acceptable error range of ±5%, mean workload from sessions 1–3 accurately anticipated the experienced workload in 55% of remaining sessions. Using the acceptable error range of ±10%, mean workload from sessions 1–3 accurately anticipated the experienced workload in 89% of remaining sessions (Figure 3).

## 4. Discussion

Our study adds to the limited body of literature quantifying the external workloads experienced by basketball players during various 5 vs. 5 games-based drills. The findings in this study provide the first insight into the ability to anticipate external workloads in basketball. We showed few monitored sessions were needed to develop reference external workload values for each drill, predicated on the typical workload completed across all sessions. External workload could only be anticipated in 0–55% of sessions when adopting an acceptable error range of ±5% during different 5 vs. 5 games-based drills. In turn, external workload was anticipated in 58–89% of sessions when using an acceptable error range of ±10% during the same drills. These outcomes provide useful insight for basketball coaching staff seeking to implement or currently implementing microsensor monitoring systems.

The ability to anticipate workload has only been investigated using internal workload measures and in team sports other than basketball. In this regard, Perrotta, Taunton, Koehle, White, and Warburton [25] reported a significant correlation (r = 0.92, *p* < 0.01) between prescribed and experienced heart rate-derived workloads in elite field hockey players over a 5-week mesocycle. Meanwhile, Lacome, Simpson, Broad, and Buchheit [26] reported a very large correlation (r = 0.78) between predicted and experienced heart rate responses in elite soccer players across an entire season. However, internal workload conceptually and practically differs from external workload and it cannot be assumed findings in existing field-based team sport research will transfer to external workload measures. Moreover, as team sport coaches prescribe training using external workloads to bring about desired internal responses in players, it is more practical to be able to anticipate external workload and perform real-time monitoring of internal workload to ensure players are responding accordingly [4]. Consequently, to our knowledge, this study is the first to anticipate player workload using external measures in team sports, providing novel outcomes specific to basketball.

We showed normative external workloads could be established using only 3–4 training sessions when using an acceptable error range of ±5% of the 95% CI for the mean workload obtained across the entire season. However, our data demonstrate relatively poor accuracy (0–55%) when using an acceptable error range of ±5% to anticipate external workload during 5 vs. 5 games-based drills. The session-to-session variation in external workload using this error range was likely due to the workload management strategies implemented by coaching staff (i.e., varying in training intensity and volume to optimize player readiness for games) [27], as well as variations in the technical and tactical approaches adopted by players (i.e., use of different offensive or defensive strategies). In addition, external workload was reported relative to training time (AU·min^−1^) to account for the varied durations of each drill across sessions. In this sense, variation in drill duration likely influenced the external workload intensities experienced by players, with basketball players shown to accomplish higher training intensities when external workload is sampled across shorter durations [28]. Consequently, our results suggest external workload cannot be accurately anticipated during 5 vs. 5 games-based drills using an acceptable error range of ±5% from normative values; however future research is encouraged to examine the influence of drill duration on anticipated workloads using conservative acceptable error ranges.

In contrast, we showed normative external workloads could be established using only 2–3 training sessions when using an acceptable error range of ±10% of the 95% CI for the mean workload obtained across the entire season. Consequently, the more liberal acceptable error range of ±10% yielded greater accuracy (58–89%) in anticipating the external workload experienced during 5 vs. 5 games-based drills. Accuracy may however be dependent upon the drill used as the number of sessions accurately anticipated was just over half (58%) for 5 vs. 5 half-court drills, which increased to 67% and 89% for 5 vs. 5 and 5 vs. 5 vs. 5 full-court drills, respectively. The greater accuracy in anticipating external workload in full-court games-based drills might be due to completion of more consistent running demands during transitions across the court than half-court games-based drills, which evoke more play sequences close to the basket underpinned by variable movement patterns [29] and technical actions [30]. These findings suggest basketball coaches can anticipate external workloads across the season in most sessions when adopting an acceptable error range of ±10%, enabling them to potentially prescribe external workloads proactively rather than retrospectively analyze and interpret data.

The present data add to the limited body of knowledge surrounding the external workloads experienced during various 5 vs. 5 games-based drills using microsensors. The mean external workloads we observed vary from results reported in previous studies. Specifically, Schelling and Torres [18] (half-court: 12.0 ± 5.6 AU·min^−1^; full-court: 17.9 ± 4.6 AU·min^−1^) and Montgomery et al. [17] (half-court: 21.0 ± 11.0 AU·min^−1^) reported higher relative external workloads than we measured during 5 vs. 5 games-based drills in professional, adult and elite, junior, male players, respectively. However, variations in accelerometer placement across studies likely accounts for these differences given past studies placed the accelerometers on the hips [18] and lumbosacral regions [17] of players, close to the center of mass. Consequently, the higher workloads reported in these studies is likely a function of the increased sensitivity to whole-body movements when positioning accelerometers closer to the center of mass [31]. In contrast, Vazquez-Guerrero et al. [19] reported values of 0.7 ± 0.1 AU·min^−1^ and 1.0 ± 0.1 AU·min^−1^ during half- and full-court 5 vs. 5 games-based drills in professional, male players. Despite these data being approximately tenfold lower than the external workloads we reported, differences in the monitoring system, algorithm, and sampling rate used by Vazquez-Guerrero et al. [19] make it difficult to conduct direct comparisons between studies.

Our study also quantified the external workload experienced during a novel 5 vs. 5 vs. 5 games-based drill. This drill is practically advantageous for basketball coaching staff as it allows implementation of short break periods, which resemble the intermittent nature of basketball game-play. Furthermore, as previously shown by Conte, Favero, Niederhausen, Capranica, and Tessitore [32], intermittent games-based drills elicit a lower %HR_max_ (*moderate*) and RPE (*small*) than continuous games-based drills. Consequently, the inclusion of short break periods permits coaches to reduce the demands imposed on players if desired by lowering the work:rest ratio encountered [32].

Although our study provides important information regarding the use of microsensors to quantify and anticipate external workload during 5 vs. 5 games-based drills in basketball, some limitations should be considered. (1) The occurrence of the included 5 vs. 5 games-based drills could not be controlled, resulting in varied sample sizes, in addition to a relatively small sample size, for analysis per drill. Nevertheless, the varied sample sizes is a consequence of conducting applied studies in actual training scenarios, which strengthens the ecological validity of our outcomes. Further, we used bootstrapping, a resampling technique, in an attempt to better estimate the parameters of workload. (2) Where drills were performed more than once in a session, only the first occurrence of the drill was utilized for analysis to control for the confounding influence of fatigue on workloads. Consequently, the inability to anticipate the external workload we observed only applies to the first occurrence of each drill in each session. (3) Drill length was not controlled for across sessions. Future research is encouraged to investigate the potential relationship between drill length and intensity during different games-based drills in basketball. (4) Given the observational nature of the present study, the timing of the included 5 vs. 5 games-based drills within each session could not be controlled. Consequently, the 5 vs. 5 games-based drills were administered ~40–56 min into each session on average. Further research is encouraged to determine whether external workload can be more accurately anticipated during games-based drills when administered at other timepoints during training sessions. (5) Player activity prior to and following each training session was not controlled. However, team training schedules remained consistent across the season, likely allowing for similar recovery and preparation leading into each session. Furthermore, we assessed the ability to anticipate external workload during games-based drills across the season in a natural team training environment to enhance the translation of findings to practical settings. (6) Acceptable error ranges of ±5% and ±10% were included in this exploratory analysis; however, some coaching staff may be more conservative or liberal in their approach, which warrants further research using alternative acceptable error ranges.

## 5. Conclusions

Normative values obtained across 3–4 sessions showed limited accuracy (0–55%) in anticipating external workload using an acceptable error range of ±5% during 5 vs. 5 games-based drills in semi-professional, male basketball players. However, normative values obtained across 2–3 sessions accurately anticipated external workload across most sessions (58–89%) using an acceptable error range of ±10% during the same drills. Consequently, external workload can be anticipated in most instances during 5 vs. 5 games-based drills using normative values established during a short-term monitoring period using an acceptable error range of ±10%.

Our findings have important implications for basketball coaching staff when implementing microsensor monitoring systems in practice. The ability to anticipate external workload using a small sample of sessions to establish normative values allows for the short-term uptake of microsensor monitoring systems, reducing the extensive financial cost, labor, and expertise associated with the long-term monitoring of players across the entire season. In addition, the normative external workloads we provided for different 5 vs. 5 games-based drills in basketball should be considered by coaches when developing training sessions across the season. The novel 5 vs. 5 vs. 5 drill examined in this study may provide a useful training stimulus for coaches when seeking to incorporate short rest periods between active play and up to 15 players in a large team environment, which is important given bench players can complete less training demands than starting players in basketball settings [33].

## Figures and Tables

**Table 1:** The format, rules and conditions used in each games-based drill.

Variables	Drill Format		
	5 vs. 5	5 vs. 5	5 vs. 5 vs. 5
Number of players	10	10	15
Duration	13.4 ± 8.9	8.0 ± 3.6	13.5 ± 7.8
Teams/players involved	2/10	2/10	3/15
Playing area	Half-court (14 m × 15 m)	Full-court (28 m × 15 m)	Full-court (28 m × 15 m)
Nature of play	Continuous	Continuous	Intermittent
Defensive Scheme	Man-to-man (switch permitted)	Man-to-man (switch permitted)	Man-to-man (switch permitted)
Free throws	Not permitted	Not permitted	Not permitted
Time-outs	Not permitted	Not permitted	Not permitted
Shot clock	24 s	24 s	24 s
Rules	To begin play, the ball was checked by the opposing team outside of the three-point line. After a point scored, turn-over or ball out-of-bounds, the ball was immediately taken to the uppermost point of the three-point line and re-checked by the now opposing team to restart play.	To begin play, the ball was checked by the opposing team outside of the three-point line. Game-play was then conducted as per normal rules.	Three teams were positioned on the court, one located on each half and one beginning with possession. To begin play, the ball was checked outside of the three-point line by the opposing team. If a point was scored, the offensive team transitioned to the other half and play was continued against the third team. In the event of a defensive rebound or ball out of bounds, the defensive team transitioned and began offensive play against the third team. No defensive pressure was applied until the ball transitioned into the opposing half of the court.
Fouls	Personal fouls were called by players on defense only. Where a foul was called, the ball was immediately thrown or taken to the uppermost point of the three-point line and checked by the opposing team to begin play.		
Out-of-bounds	Throw-ins were not permitted. If the ball was to go out-of-bounds, the ball was immediately replaced, brought to the uppermost point of the three-point line and checked by the opposing team to restart play.		
Technical/tactical task	No specific technical or tactical task was provided by the coaching staff		
External motivation	Verbal encouragement was provided to the players by the coaching staff		
